# Feasibility of energy expenditure estimation during outdoor walking using multimodal in-ear sensors: a preliminary validation study against indirect calorimetry

**DOI:** 10.1038/s41598-026-64756-6

**Published:** 2026-08-01

**Authors:** David Camargo, Incinur Zellhuber, Michael Weber, Martin Schönfelder

**Affiliations:** 1 Department of Health and Sport Sciences, Exercise Biology, TUM School of Medicine and Health, Technical University of Munich, Munich, Germany; 2 Cosinuss GmbH, Munich, Germany

**Keywords:** In-ear sensor, Wearable, Energy expenditure, Heart rate, Accelerometry, Outdoor walking, Physical activity, Indirect calorimetry, Feasibility study, Participant-calibrated prediction, Health care, Physiology

## Abstract

A growing number of portable devices has become available for daily physical activity and energy expenditure (EE) monitoring. Although several studies have examined EE estimation using heart rate (HR) and accelerometry (ACC), no study to date has explored EE prediction based on HR and ACC data measured simultaneously in the ear canal by a single commercial wearable device. Therefore, the aim of this study was to assess the feasibility of estimating EE using the cosinuss$^\circ$ in-ear sensors $^\circ$One and $^\circ$Two. Twenty-four healthy adults (12 female, 12 male, age 26.91 ± 2.91 years, body mass 65.43 ± 8.01 kg) completed two test sessions: a laboratory treadmill test (Modified Bruce Protocol) and an outdoor walking test consisting of three phases (uphill at comfortable pace, downhill at comfortable pace, and uphill as fast as possible). EE was measured by the portable indirect calorimetry device MetaMax 3B (criterion measure), while HR and ACC were recorded continuously by the in-ear sensors $^\circ$One and $^\circ$Two. Linear mixed-effects models were developed from laboratory data and applied to outdoor walking data from the same participants (participant-calibrated prediction). Prediction accuracy was assessed using root mean square error (RMSE), mean absolute percentage error (MAPE), and Bland-Altman analysis. Both in-ear sensors showed preliminary potential for EE prediction during outdoor walking. The integration of HR, ACC, and biological sex produced the best-fitting calibration models, with marginal $R^2$ values of 0.79 and 0.80 for $^\circ$One and $^\circ$Two respectively. In the outdoor walking test, no significant systematic bias was detected across conditions (*p> 0.05*). However, prediction accuracy was variable across conditions and subgroups, with MAPE ranging from 22% to 37% for $^\circ$One and 22% to 38% for $^\circ$Two (following exclusion of one participant with implausible reference data in the Down phase). Pearson correlation coefficients were stronger for female participants, while male subgroup correlations were weak or negative in several conditions, limiting conclusions about individual-level prediction accuracy for this subgroup. The cosinuss$^\circ$ in-ear sensors demonstrated preliminary feasibility for group-level EE estimation during outdoor walking, with accuracy broadly comparable to several commercial wrist-worn devices. Given the variable prediction accuracy, particularly for male participants and during downhill walking, further validation with larger and more diverse samples is needed before clinical or sports application.

## Introduction

Energy expenditure (EE), except during non-steady state activities, is the result of converting energy substrates into usable energy through a complex process including oxygen consumption^1^. The total EE in humans comprises basal metabolic rate, thermic effect of food and physical activity-related energy expenditure^2^. The physical activity-related EE measures energy expended in physical activities and varies significantly from person to person due to factors like body weight, lifestyle choices and exercise efficiency^3^. Additionally, differences in EE between sexes have been observed, which can be attributed to significant variations in lean body mass between women and men^4^. The exercise EE is critical for improving the life quality for an aging population, as it is modifiable through regular physical activity and exercise, contributing to individuals’ cardiovascular health, muscle strength, and weight regulation^5^.

Monitoring of physical activity and its associated EE has become important in clinical treatment plans, especially for chronic diseases such as chronic obstructive pulmonary disease^6^, or for postoperative patients to prevent further complications^7^. Therefore, there is a growing interest in objective, accurate and reliable measurement of physical activity related EE on an individual basis.

Measurement of energy expenditure involves various methods, from direct calorimetry techniques to estimation-based methods, including physiological and kinematic variables^8,9^. The gold standard for measuring exercise EE is indirect calorimetry, which evaluates oxygen consumption ($\dot{V}$O$_2$) and carbon dioxide production ($\dot{V}$CO$_2$)^10,11^, typically using the Weir equation during physical activities^11^. Although many modern indirect calorimetry systems are designed as portable metabolic devices, allowing field-based measurements^12^, they are still limited to laboratory or clinical settings due to the equipment and expertise required for accurate measurements.

Previous research has shown that physiological and kinematic variables, such as HR and body acceleration (ACC), can also be used to predict EE^2,13,14^. HR is related to oxygen uptake and consumption in exercise physiology, regardless of age, sex, or the presence of various disease states, and is thus closely linked to EE^10,15,16^. The relationship between HR and EE has been shown to be linear during dynamic muscle work at low to moderate intensities, up to approximately 65% of the individual’s maximal heart rate^14,17^. Beyond this threshold, the relationship becomes curvilinear. However, HR alone is not sufficient for accurate EE estimation at low activity levels^18,19^.

Similarly, a linear relationship has been reported between EE and body acceleration in walking^20–23^. In 2001, Strath et al^18^. proposed a method for estimating EE by integrating ACC and HR data (ACCHR), demonstrating that this combined approach outperforms the use of either metric alone. This was later substantiated by Brage et al^24^., who demonstrated that integration of HR and ACC significantly improves both accuracy and precision of EE estimations. Subsequent studies validated the ACCHR method across various physical activities, including uphill running^25,26^, and high-intensity exercise such as Tabata^27^.

While several studies have investigated multi-sensor wearable devices measuring both HR and ACC, almost all are worn on the wrist, chest, or hip^28–32^. Two studies have examined ear-worn sensors for EE prediction, in adult populations, including healthy and clinical groups, but both used accelerometer-only devices that are not commercially available^33,34^. To our knowledge, no study has yet demonstrated the potential of a multimodal in-ear sensor, capable of measuring HR and ACC simultaneously, for accurate estimation of EE.

The ear offers several anatomical advantages for wearable sensing. These anatomical and physiological properties make the ear canal a promising site for continuous physiological monitoring under free-living and exercise conditions. In this context, in-ear sensors offer a practical alternative to more obtrusive torso- or limb-worn devices. Ear-mounted devices are less obtrusive, promoting sustained wear and improving user compliance. The anatomical stability of the ear minimises motion artifacts common in peripheral sensor locations, contributing to improved signal quality. Recent studies have identified the ear as a promising site for gait monitoring and integrated vital sign monitoring^35,36^.

While the present feasibility study is restricted to healthy young adults, future work will need to establish the reproducibility of these findings across more diverse populations. Therefore, the purpose of this study was to examine the validity of the cosinuss$^\circ$ in-ear sensors $^\circ$One and $^\circ$Two for estimating EE, based on HR and ACC recordings, compared with the MetaMax 3B indirect calorimetry device (criterion measure), during treadmill and outdoor walking at different paces. Additionally, the study aimed to compare sensor performance between male and female participants.

## Methods

### Participants

Twenty-four healthy adults (12 females and 12 males), between the age of 20 and 35, volunteered to participate in this study (Table 1). Before the tests, participants were informed about the study details, potential risks and aims, and signed a written informed consent form. Participants without any disability in the lower limbs were included. Exclusion criteria were current or previous injury in the lower extremity or lower back, or any condition affecting leg muscle activation.

Twenty-four participants completed both the laboratory and outdoor sessions. For the outdoor testing phases, the number of valid participants varied per phase and per sensor due to intermittent data loss from Wi-Fi or Bluetooth disconnections during individual phases. Exclusions were applied independently per sensor and per phase; participants with complete data loss in a given phase were excluded from that phase only and retained in phases where data were intact. The resulting sample sizes for sensor $^\circ$One were: Up phase *n = 12*, Down phase *n = 12*, Up Fast phase *n = 11*. For sensor $^\circ$Two: Up phase *n = 13*, Down phase *n = 10* (following exclusion of one participant with implausible reference data; see Data Analysis), Up Fast phase *n = 9*. All per-phase sample sizes are reported in Tables 3 and 4.

**Table 1:** Participant characteristics (mean ± SD).

Variable	All (n=24)	Male (n=12)	Female (n=12)
Age (yr)	26.91 (±2.91)	26.5 (±2.75)	27.33 (±3.00)
Height (cm)	173.54 (±6.89)	176.08 (±5.92)	171.00 (±6.86)
Weight (kg)	65.43 (±8.01)	70.35 (±6.10)	60.52 (±6.55)
BMI (kg*·*m$^{-2}$)	21.68 (±1.91)	22.67 (±1.43)	20.69 (±1.83)
Estimated $\dot{V}O_{2\text {max}}$ (mL*·*kg$^{-1}\cdot$min$^{-1}$)	44.27 (±9.25)	44.23 (±11.47)	44.31 (±6.31)

Participants’ cardiorespiratory fitness was characterised as estimated $\dot{V}O_{2\text {max}}$ derived from the final stage of the Modified Bruce Protocol using standard prediction equations; the test was terminated at 85% of age-predicted maximal heart rate and therefore does not represent directly measured $\dot{V}O_{2\text {max}}$.

Of the 24 participants who completed the laboratory condition, data from four participants were excluded from the calibration analysis for sensor $^\circ$One (participants 1, 4, 8, and 19) and four participants for sensor $^\circ$Two (participants 4, 7, 11, and 16), due to data loss resulting from Bluetooth disconnection or signal dropout during the laboratory recording. All 24 participants contributed data to the outdoor validation phase. Exclusions were applied independently per sensor, resulting in 20 participants used for the $^\circ$One calibration model and 20 participants for the $^\circ$Two calibration model.

The ethics committee of the Technical University of Munich approved the study protocol (No. 2022-558-SSR). The study was conducted in accordance with the principles of the Declaration of Helsinki.

### Sample size and power analysis

An a priori power analysis was conducted using G*Power (version 3.1.9.7). For bivariate correlation analysis between predicted and measured EE, a minimum sample size of 21 was required to detect a large effect size (*ρ = 0.5*) with 80% power (*α = 0.05*), based on effect sizes reported in previous wearable sensor validation studies examining combined HR and accelerometry against indirect calorimetry^18,37^. For multiple regression, a sample of 31 was estimated for a large effect ($f^2 = 0.35$). Although the final sample of 24 participants fell below the regression target, it exceeded the minimum threshold for correlation analysis and was considered sufficient for the mixed-effects calibration given the repeated-measures structure of the laboratory data, in which multiple steady-state observations per participant were available for model fitting.

### Recording devices

#### In-ear sensors °One and °Two

**Fig. 1:**
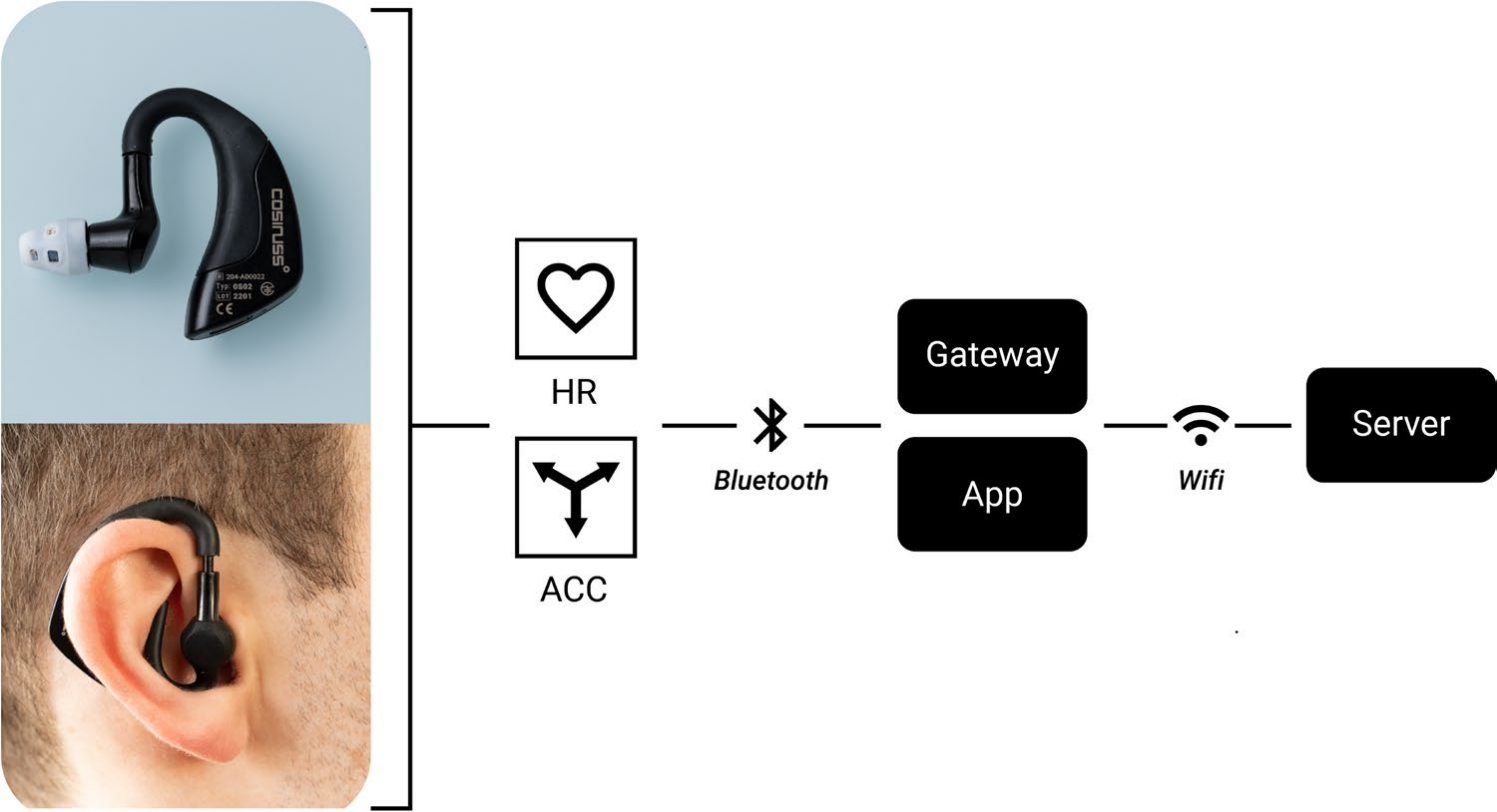
Scheme of the cosinuss$^\circ$ data collection setup. From left to right: the in-ear sensor ($^\circ$One and $^\circ$Two are represented by the same sensor image) and an illustrative depiction of a person wearing the sensor in the right ear. HR and ACC data are transferred from the in-ear sensors to the cosinuss$^\circ$ gateway or mobile app via Bluetooth Low Energy, and then to the server via Wi-Fi.

The in-ear wearable sensors used in this study were $^\circ$One and $^\circ$Two (Cosinuss GmbH, Munich, Germany) (Figure 1). Both sensors are professional fitness trackers, having a size of 45 *×* 38 *×* 18 mm and a weight of 6.5 grams. The silicon sensor head, including a resistance temperature sensor and an optical sensor for photoplethysmography (PPG), is placed in the outer ear canal and stays in close contact with the skin. The main difference between the two sensors is that $^\circ$One uses a green light-emitting diode (LED) with a wavelength of 520 nm, while $^\circ$Two uses red and infrared LEDs with wavelengths of 665 nm and 940 nm for PPG. Heart rate is derived from the PPG signals of both sensors directly on the sensor, with a sampling rate of 1 Hz.

The in-ear sensors also include a 3D-axis accelerometer, which computes the sum of inertial linear and gravitational accelerations along its axes. Because this study aimed to determine whether a single device could effectively predict energy expenditure, both HR and ACC data from the in-ear sensor were utilised.

#### Polar H7 chest strap

The Polar H7 (Polar Electro, Kempele, Finland) was used to assess HR throughout the study, serving as a reference for the in-ear HR measurements. The Polar H7 monitors HR based on electrocardiography (ECG) and has been validated against the ECG Holter gold standard^38^.

#### MetaMax 3B

The MetaMax 3B portable, breath-by-breath indirect calorimetry system (Cortex Biophysik GmbH, Leipzig, Germany) was used as the EE measurement criterion. The MetaMax 3B has been shown to reliably measure metabolic demands and has been used as a reference for wearables designed to monitor EE in previous studies^9,29,39^. EE was calculated using the Weir equation:

1 $$\begin{aligned} \text {EE (kcal/min)} = 3.941 \times \dot{V}\text {O}_2\text {(L/min)} + 1.106 \times \dot{V}\text {CO}_2\text {(L/min)} \end{aligned}$$

With VO$_2$ and VCO$_2$ expressed in L/min, the Weir equation yields EE in kcal/min. Values were subsequently multiplied by 60 to convert to kcal/h for all analyses and reporting.

### Study protocol

Participants were asked not to ingest alcohol or caffeine for 24 hours and to abstain from food for 3 hours before the tests to reduce the thermic effect of food. The study protocol was divided into two parts: laboratory and outdoor tests.

#### Laboratory tests

The in-ear sensors $^\circ$One and $^\circ$Two were placed in the left and right ears, respectively. The Polar H7 chest strap and the MetaMax 3B were placed according to device usage guidelines. After equipment fitting, participants’ height and weight were measured. Participants then performed a submaximal treadmill walking test using the Modified Bruce Protocol^40,41^, which involves walking on a treadmill with speed and incline increasing every three minutes (up to a maximum of seven stages).

The test was terminated when the participant reached 85% of age-predicted maximum heart rate (HRmax *= 208 - 0.7 ×* age)^42^, or when it was deemed unsafe or inappropriate to continue at the investigator’s discretion. In cases where HR transiently exceeded 85% HRmax during a stage transition but returned below threshold before the end of that stage, the test continued.

#### Outdoor tests

Three days after the laboratory measurements, participants conducted the second part of the study outdoors. Outdoor measurements consisted of walking a one-kilometre path uphill at a comfortable pace, waiting five minutes at the top, then walking the same path downhill at a comfortable pace. After five minutes, participants walked the same path uphill as fast as possible. The location was Olympiaberg, a 60-metre hill in Olympiapark, Munich, with an average gradient of 4.6%.

### Data acquisition

Each participant was fitted simultaneously with the in-ear sensors $^\circ$One (left ear) and $^\circ$Two (right ear), the Polar H7 chest strap, and the MetaMax 3B. Throughout the protocol, the Polar H7 measured HR based on ECG and transmitted data to the MetaMax 3B via Bluetooth 4.2. The in-ear sensors were connected to the cosinuss$^\circ$ mobile app and gateway via Bluetooth 4.2, with data transferred to the cosinuss$^\circ$ server via Wi-Fi. Acceleration was measured at 100 Hz with accuracy of ± 0.002 m/s$^2$. The three-axis signals were combined into a single measure by calculating the vector magnitude $\Vert a\Vert = \sqrt{x^2 + y^2 + z^2}$. To remove the gravitational component, 1 g was subtracted from the magnitude, and the absolute deviation from 1 g, $\left| \Vert a\Vert - 1 \right|$, was used as the acceleration variable. Data was expressed in units of g, and no additional filtering or smoothing was applied; values were averaged over the analysis window. HR was recorded at approximately 1 Hz with accuracies of ± 1 bpm ($^\circ$One) and ± 2 bpm ($^\circ$Two).

### Data analysis

#### Preprocessing

HR and ACC data were downloaded from the cosinuss$^\circ$ server for offline analysis. Microsoft Excel was used for visual inspection of HR trace alignment only; all quantitative analyses were conducted in Python 3.12. For synchronisation, HR data from the Polar H7 and in-ear sensors were plotted together to confirm temporal alignment; no time offset was detected requiring correction. Downsampling was performed based on the MetaMax 3B file of each participant using MATLAB (Version 2020a), ensuring equal sample sizes across devices. The MetaMax 3B was calibrated according to manufacturer specifications before each test session. Breath-by-breath data were smoothed using a 15-breath rolling average. Epochs with abrupt VO$_2$ changes exceeding 30% between consecutive breaths were flagged and excluded as artefacts prior to analysis. A reference EE exclusion criterion was defined a priori: participant sessions with mean reference EE below 150 kcal/h during an outdoor walking phase were excluded as indicative of signal artefact in the reference device.

#### Statistical analysis

Before finalising the models, we evaluated multiple linear regression using polynomial and exponential terms; however, neither offered meaningful improvements in predictive accuracy over a mixed-effects framework. Consequently, Linear Mixed-Effects Models (LMM) were developed to account for inter-individual metabolic variability and the clustered structure of repeated measurements within participants. To ensure physiological stability for model calibration, a 30-second steady-state window (150–180 s) was extracted from the end of each Bruce protocol stage, ensuring that both HR and oxygen consumption had reached a plateau before analysis.

The models incorporated HR, ACC, and biological sex as fixed effects. Sex was coded as a binary variable (0 *=* Male, 1 *=* Female). A random intercept was included for each participant to control for individual differences in baseline metabolism. A sensitivity analysis was performed for each sensor by comparing the full model against simpler iterations (HR-only, ACC-only, and HR+ACC without sex). The optimal models were selected based on highest marginal explanatory power during calibration. To maintain consistency between sensor models and allow direct comparison of results across $^\circ$One and $^\circ$Two, the same fixed-effect structure (HR, ACC, Sex) was applied to both sensors, regardless of individual predictor significance within each model.

For the outdoor condition, sensor signals and MetaMax 3B reference data were temporally aligned in Python. To address the dependent nature of the data and prevent overrepresentation of individual participants, a single estimate was calculated per participant by averaging the measured and predicted EE across the steady-state period of each outdoor test phase. Predictions were generated using the full LMM including participant-specific random intercepts estimated during calibration. Because outdoor data were collected from the same participants as the calibration dataset, this constitutes participant-calibrated prediction rather than independent external validation. Generalisation to new, unseen participants would require fixed-effect-only prediction; the present analysis therefore reflects the upper bound of expected performance and should be interpreted accordingly. The steady-state period was identified based on visual inspection of the HR and EE traces confirming physiological stabilisation. These participant-level averages were then used for all validation statistics.

Statistical analysis was performed using Python (Version 3.12). The LMM was implemented using the statsmodels library. For the calibration phase, model fit and explanatory power were assessed using Nakagawa’s coefficient of determination, reporting both the marginal $R^2$ ($R^2_m$), representing variance explained by the fixed effects (ACC, HR, Sex), and the conditional $R^2$ ($R^2_c$), representing the total variance explained by both fixed and random effects. Nakagawa’s $R^2$ was applied exclusively to the calibration phase, where the LMM structure is appropriate given the repeated-measures design.

For the outdoor testing phase (participant-calibrated prediction using the same participants as calibration), model accuracy was assessed using RMSE and MAPE. Agreement between predicted and reference EE was assessed using Bland-Altman analysis^43,44^. Two-tailed one-sample *t*-tests were used to determine whether the mean difference (bias) differed significantly from zero. To explore the relationship between predicted and actual EE in the validation phase, Pearson correlation coefficients were calculated using participant-level averaged values and treated as descriptive given the small sample sizes. This approach is appropriate because only one averaged EE estimate per participant was available for the validation phase, making the repeated-measures LMM framework unsuitable at this stage.

To compare prediction accuracy and physiological characteristics between sexes, two-tailed independent Welch’s *t*-tests were used to compare mean predicted EE, reference EE, RMSE, and MAPE between male and female participants for each outdoor phase. Given the small subgroup sizes, comparisons of Pearson correlation coefficients between sexes were treated as descriptive rather than inferential.

Heart rate accuracy of both in-ear sensors was assessed by comparing sensor HR against the Polar H7 chest strap reference using RMSE, mean difference, limits of agreement, and Pearson correlation, calculated from steady-state observations in the laboratory calibration dataset.

The significance level for all analyses was set at *p < 0.05*. All data visualisations were generated using Matplotlib.

To ensure computational efficiency and code optimisation, a Large Language Model (Claude; Anthropic, San Francisco, CA) was utilised to assist with code refinement and Python syntax suggestions. All analytical logic, model specification, statistical decisions, and final code were designed, verified, and executed by the authors. No analytical outputs were accepted without manual verification against raw data.

### AI assisted copy editing

Generative AI tools (specifically large language models) were used for code refinement (syntax suggestions) and for grammatical and stylistic copy-editing of the manuscript. These tools did not generate scientific content, perform data analysis, or contribute to data interpretation or conclusions. All analytical methods, model specifications, and code were developed and verified by the authors, with results cross-checked against the raw data. All scientific content was written and critically reviewed by the authors to ensure accuracy.

## Results

### Laboratory condition

Twenty-four participants completed the laboratory condition without adverse effects. For both in-ear sensors, the model combining HR, ACC, and Sex as fixed effects provided the best fit. The equations derived from calibration, subsequently applied to the outdoor validation data, are:

2 $$\begin{aligned} \text {EE (kcal/h)} = -296.505 + (403.746 \cdot \text {ACC}) + (6.002 \cdot \text {HR}) + (-90.821 \cdot \text {Sex}) \quad (^\circ \text {One}) \end{aligned}$$

3 $$\begin{aligned} \text {EE (kcal/h)} = -378.965 + (140.967 \cdot \text {ACC}) + (6.958 \cdot \text {HR}) + (-38.692 \cdot \text {Sex}) \quad (^\circ \text {Two}) \end{aligned}$$

In both equations, Sex was coded as a binary variable (0 *=* Male, 1 *=* Female), such that female participants receive a negative adjustment to predicted EE reflecting the lower absolute EE captured in the calibration data.

The explanatory power of the calibration models was assessed using Nakagawa’s coefficient of determination. For sensor $^\circ$One, the fixed-effect formula alone explained 78.8% of the variance ($R^2_m = 0.7883$). When accounting for individual metabolic baselines, the total model fit reached 92.1% ($R^2_c = 0.9210$), with a group variance (random intercept) of 10,916.6 and residual variance of 6,496.4.

For sensor $^\circ$Two, the formula explained 80.4% of the variance ($R^2_m = 0.8035$). The total model fit was 94.7% ($R^2_c = 0.9468$), supported by a group variance of 11,453.7 and a residual variance of 4,252.6.

A sensitivity analysis was performed for both sensors to evaluate the contribution of individual predictors (Table 2). For sensor $^\circ$One, removing the biological sex covariate reduced the marginal explanatory power to $R^2_m = 0.7719$, while models using only HR ($R^2_m = 0.7269$) or only ACC ($R^2_m = 0.5978$) showed lower predictive accuracy, confirming that the full model provided the best calibration fit. For sensor $^\circ$Two, a similar pattern was observed for HR-only ($R^2_m = 0.7936$) and ACC-only ($R^2_m = 0.6631$) models; however, the ACC + HR model without sex ($R^2_m = 0.8050$) marginally exceeded the full model ($R^2_m = 0.8035$), suggesting that sex did not meaningfully improve the $^\circ$Two calibration model beyond HR and ACC alone.

**Fig. 2:**
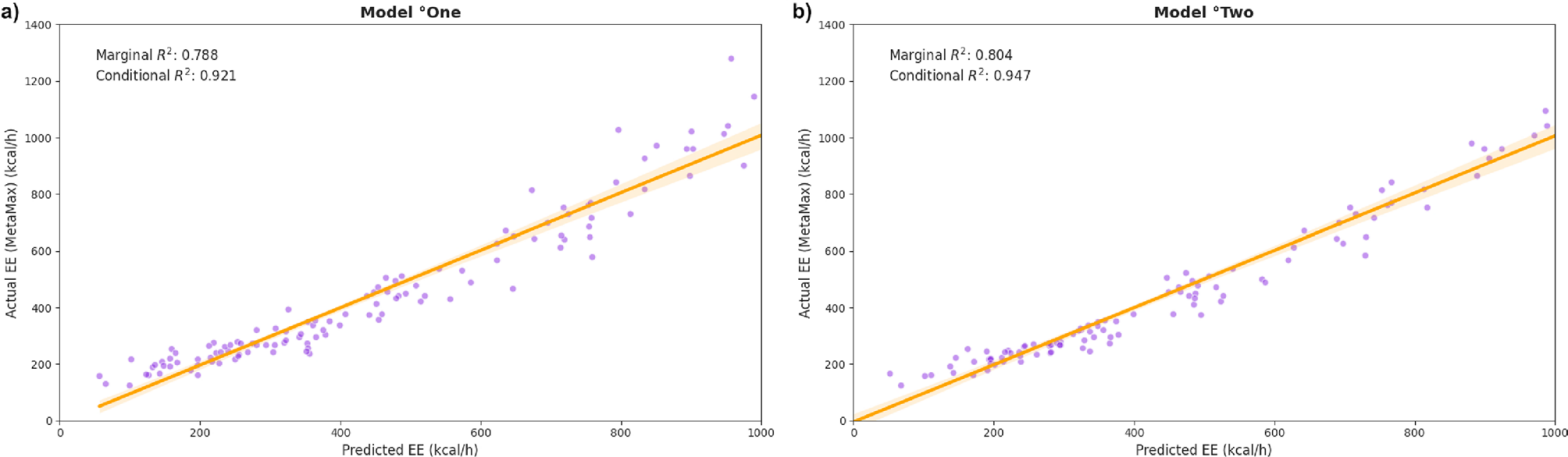
Linear Mixed Models (LMM) for energy expenditure prediction: Comparison between predicted EE from in-ear sensors $^{\circ }$One (**a**) and $^{\circ }$Two (**b**) versus reference measurements from MetaMax 3B. Nakagawa marginal $R^{2}$ represents the variance explained by fixed effects, while Nakagawa conditional $R^{2}$ represents the variance explained by the entire model (fixed and random effects).

### Outdoor condition

#### In-ear sensor °One

**Fig. 3:**
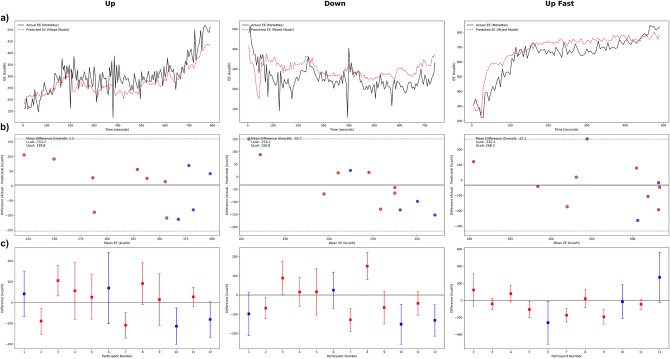
Combined results from sensor $^\circ$One. (**a**) Epoch-by-epoch model output for participant 11, illustrating the temporal tracking of predicted versus measured EE across the three outdoor phases. Note: statistical comparisons used a single participant-level average per phase, not the epoch-level values shown here. (**b**) Bland-Altman plots for all participants for each outdoor phase. Dashed lines show 95% limits of agreement (mean difference ± 1.96 SD). Blue: male; Red: female. (**c**) Individual mean differences with standard deviation per participant.

Examining the individual fixed-effect coefficients in the $^\circ$Two model, only HR was a statistically significant predictor (*p < 0.001*), while ACC (*p = 0.233*) and Sex (*p = 0.440*) did not reach significance. This suggests that HR was the primary driver of EE prediction in the $^\circ$Two calibration model, and the contributions of ACC and Sex should be interpreted with caution. In contrast, for sensor $^\circ$One, HR (*p < 0.001*) and ACC (*p = 0.001*) were both statistically significant, while Sex approached but did not reach significance (*p = 0.061*).

Figure 3a illustrates the continuous epoch-by-epoch model output for a representative participant (participant 11), provided to visualise the temporal tracking of predicted versus measured EE throughout each outdoor phase. For all statistical comparisons, a single participant-level average was computed across the steady-state period of each phase, as described in the Methods.

Bland-Altman plots (Figure 3b) illustrate the agreement between predicted EE from sensor $^\circ$One and measured EE from the MetaMax 3B for each outdoor test phase. The model showed no significant bias across experimental conditions (Table 3). For the first phase (Up), the mean difference was 3 kcal/h (*p = 0.889*). In the second phase (Down), the mean difference was *-25* kcal/h (*p = 0.362*), indicating a non-significant tendency to underestimate EE. Note: a negative mean difference in Table 3 indicates overestimation by the sensor (Reference − Predicted *< 0*). No significant trends were observed in any outdoor phase. The limits of agreement were widest during high-intensity activity (Up Fast), with a 600 kcal/h discrepancy between upper and lower limits for the total group. The majority of participants had individual standard deviations below 100 kcal/h (Figure 3c).

Pearson correlation coefficients between participant-level averaged predicted and actual EE are reported in Table 3. Correlations were generally stronger for female participants, particularly in the Up Fast phase (*r = 0.83*, *p = 0.011*). Male subgroup correlations were weak or negative across all phases (Up: *r = -0.96*; Down: *r = -0.02*; Up Fast: *r = -0.50*), which should be interpreted with caution given the very small male subgroup sizes (n *= 3*). Prediction accuracy (RMSE) was lower for females in the high-intensity Up Fast phase (114 kcal/h) compared to males (218 kcal/h). The Down phase showed the highest MAPE overall (37%), with males reaching 45% and females 34%, reflecting the challenges of predicting EE during downhill locomotion with a model trained exclusively on uphill data. Overall, MAPE ranged from 22% to 37% across conditions for sensor $^\circ$One.

#### In-ear sensor °Two

**Fig. 4:**
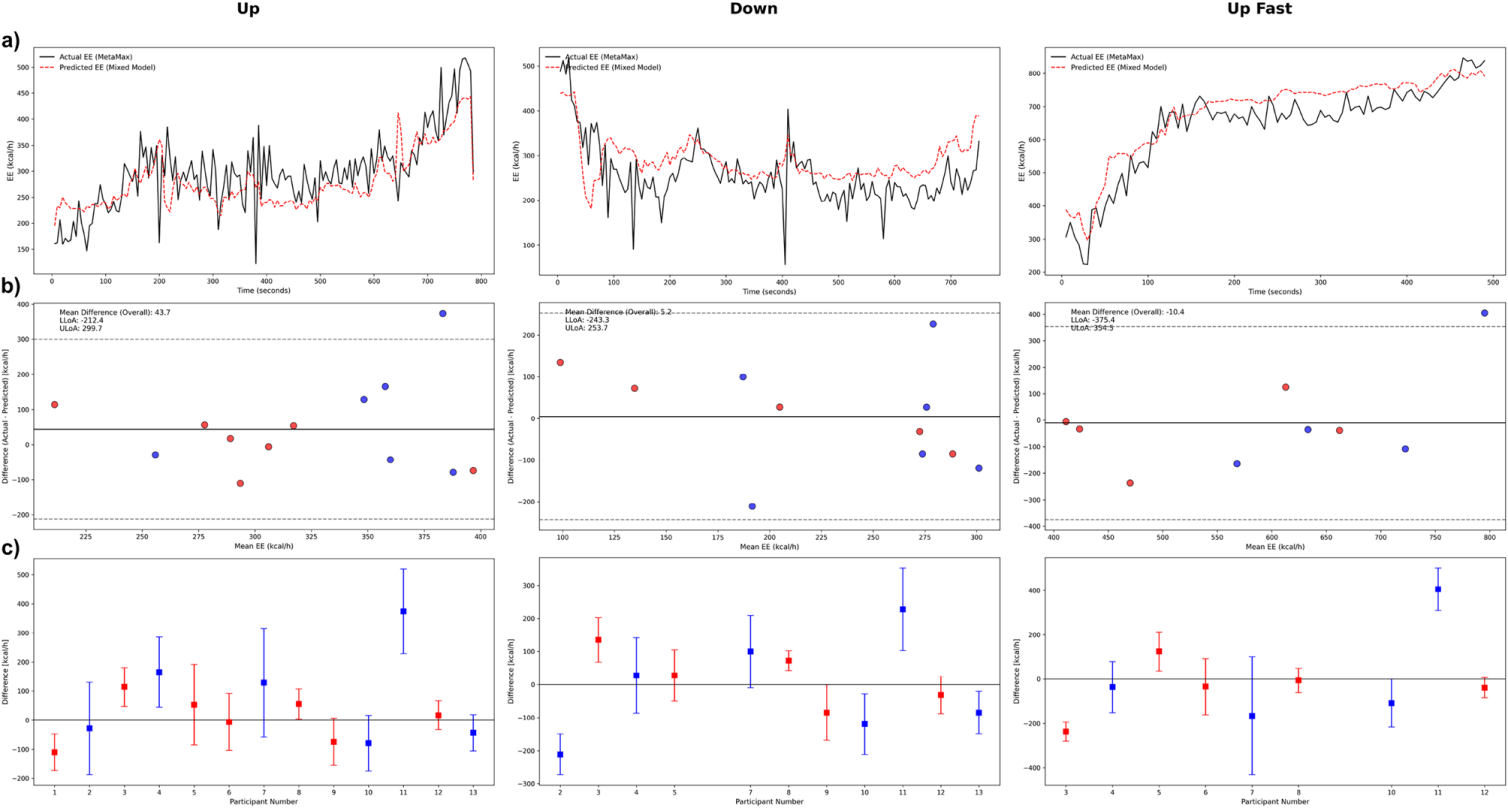
Combined results from sensor $^\circ$Two. (**a**) Epoch-by-epoch model output for participant 12, illustrating the temporal tracking of predicted versus measured EE across the three outdoor phases. Note: statistical comparisons used a single participant-level average per phase, not the epoch-level values shown here. (**b**) Bland-Altman plots for all participants for each outdoor phase. Dashed lines show 95% limits of agreement. Blue: male; Red: female. (**c**) Individual mean differences with standard deviation per participant.

Figure 4a illustrates the continuous epoch-by-epoch model output for a representative participant (participant 12). For all statistical comparisons, a single participant-level average was computed across the steady-state period of each phase, as described in the Methods.

Bland-Altman analysis (Table 4, Figure 4b) confirmed no significant systematic bias across any experimental condition (*p> 0.05*). The mean differences were small: 43 kcal/h for Up (*p = 0.252*), 5 kcal/h for Down (*p = 0.894*), and *-10* kcal/h for Up Fast (*p = 0.871*). Although the widest limits of agreement were observed in the Up Fast phase, the majority of participants maintained individual standard deviations below 100 kcal/h (Figure 4c).

Pearson correlation coefficients are reported in Table 4. One participant (P2) was excluded from the ES2 Down phase analysis due to implausible MetaMax reference EE values (mean: 85.9 kcal/h, range: 4.1–578.3 kcal/h), indicative of a signal artifact in the reference device during that phase. Following this exclusion (n *= 10*), the Down phase showed a mean difference of 27 kcal/h (*p = 0.460*), RMSE of 108 kcal/h, and MAPE of 38%. The Down phase still exhibited the highest MAPE across conditions, likely due to the lower absolute EE during descent amplifying percentage errors, combined with the absence of downhill data in model training. Male subgroup correlations were weak or negative across all phases (Up: *r = -0.55*; Down: *r = -0.47*; Up Fast: *r = -0.41*), consistent with the pattern observed for sensor $^\circ$One, and should be interpreted with caution given small subgroup sizes (n *= 4*–6).

The reference data confirmed that female participants consistently expended less energy than males during uphill walking; the model captured this physiological distinction, with predicted EE values reflecting the sex-based trends observed in the MetaMax 3B data. This finding supports the inclusion of Sex as a fixed effect in the $^\circ$One model; however, the sensitivity analysis indicated that Sex did not meaningfully improve the marginal explanatory power of the $^\circ$Two calibration model (full model $R^2_m = 0.8035$ vs ACC + HR only $R^2_m = 0.8050$), and its contribution to this model should be interpreted with caution.

While male participants showed higher predicted and reference EE values than female participants across uphill conditions, walking speed was not controlled or measured during the outdoor tests. Therefore, observed sex differences in EE cannot be attributed solely to physiological factors, as males may have walked faster than females, particularly during the Up Fast phase.

**Table 2:** Sensitivity analysis: Marginal $R^2$ ($R^2_m$) for different model specifications during calibration.

Model	Sensor 1 $R^2_m$	Sensor 2 $R^2_m$
ACC + HR + Sex (full model)	0.7883	0.8035
ACC + HR (no Sex)	0.7719	0.8050
HR only	0.7269	0.7936
ACC only	0.5978	0.6631

*Note:* For Sensor 2, the ACC+HR model without sex marginally exceeded the full model, suggesting sex did not improve the Sensor 2 calibration beyond ACC and HR alone.

**Table 3:** Statistical analysis summary and Bland-Altman comparison by sex for Sensor $^\circ$One.

Phase	Statistic	All	Male	Female
Up	n	12	3	9
	Mean Diff [95% CI] (kcal/h)	3 [*-47*, 54]	*-42* [*-284*, 201]	18 [*-38*, 75]
	*p*-value	0.889	0.536	0.475
	LLA [95% CI] (kcal/h)	*-153* [*-243*, *-64*]	*-233* [*-711*, 245]	*-125* [*-225*, *-25*]
	ULA [95% CI] (kcal/h)	160 [71, 249]	150 [*-328*, 628]	162 [62, 262]
	Mean Predicted [SD] (kcal/h)	328 (±82)	397 (±47)	304 (±79)
	EE MetaMax [SD] (kcal/h)	331 (±53)	355 (±52)	323 (±54)
	RMSE (kcal/h)	77	90	72
	MAPE (%)	22	26	21
	Pearson *r*	0.36	$-0.96^{\dagger }$	0.44
Down	n	12	3	9
	Mean Diff [95% CI] (kcal/h)	*-25* [*-83*, 33]	*-86* [*-328*, 155]	*-5* [*-70*, 61]
	*p*-value	0.362	0.263	0.874
	LLA [95% CI] (kcal/h)	*-205* [*-307*, *-102*]	*-277* [*-752*, 199]	*-172* [*-288*, *-55*]
	ULA [95% CI] (kcal/h)	154 [52, 257]	104 [*-371*, 579]	163 [46, 279]
	Mean Predicted [SD] (kcal/h)	243 (±105)	319 (±96)	218 (±101)
	EE MetaMax [SD] (kcal/h)	218 (±35)	232 (±15)	214 (±39)
	RMSE (kcal/h)	91	117	81
	MAPE (%)	37	45	34
	Pearson *r*	0.54	$-0.02^{\dagger }$	0.56
Up Fast	n	11	3	8
	Mean Diff [95% CI] (kcal/h)	*-32* [*-135*, 71]	*-4* [*-666*, 658]	*-43* [*-137*, 52]
	*p*-value	0.502	0.983	0.319
	LLA [95% CI] (kcal/h)	*-332* [*-514*, *-151*]	*-526* [*-1831*, 778]	*-264* [*-433*, *-95*]
	ULA [95% CI] (kcal/h)	268 [87, 450]	519 [*-786*, 1823]	179 [10, 347]
	Mean Predicted [SD] (kcal/h)	545 (±193)	590 (±208)	528 (±199)
	EE MetaMax [SD] (kcal/h)	513 (±138)	586 (±93)	486 (±147)
	RMSE (kcal/h)	150	218	114
	MAPE (%)	26	34	23
	Pearson *r*	0.61*	$-0.50^{\dagger }$	0.83**

* *p < 0.05*; ** *p < 0.01*; $^{\dagger }$ interpret with caution (*n = 3*). A negative mean difference indicates sensor overestimation (Reference − Predicted *< 0*). Subgroup analyses are exploratory due to small sizes (*n = 3*–9). Marginal and Conditional $R^2$ were omitted from validation results; participant-level Pearson *r* is reported instead.

**Table 4:** Statistical analysis summary and Bland-Altman comparison by sex for Sensor $^\circ$Two.

Phase	Statistic	All	Male	Female
Up	n	13	6	7
	Mean Diff [95% CI] (kcal/h)	43 [*-35*, 123]	86 [*-94*, 267]	7 [*-65*, 80]
	*p*-value	0.252	0.275	0.815
	LLA [95% CI] (kcal/h)	*-212* [*-351*, *-74*]	*-251* [*-579*, 77]	*-146* [*-276*, *-16*]
	ULA [95% CI] (kcal/h)	300 [161, 438]	423 [95, 752]	161 [30, 291]
	Mean Predicted [SD] (kcal/h)	300 (±82)	306 (±84)	295 (±86)
	EE MetaMax [SD] (kcal/h)	344 (±90)	392 (±111)	302 (±41)
	RMSE (kcal/h)	133	179	73
	MAPE (%)	26	30	22
	Pearson *r*	*-0.15*	$-0.55^{\dagger }$	0.42
Down$^{\ddagger }$	n	10	5	5
	Mean Diff [95% CI] (kcal/h)	27 [*-52*, 106]	30 [*-145*, 205]	24 [*-83*, 131]
	*p*-value	0.460	0.659	0.571
	LLA [95% CI] (kcal/h)	*-189* [*-328*, *-50*]	*-246* [*-569*, 77]	*-145* [*-342*, 52]
	ULA [95% CI] (kcal/h)	243 [103, 382]	306 [*-17*, 629]	192 [*-5*, 389]
	Mean Predicted [SD] (kcal/h)	218 (±110)	248 (±96)	188 (±126)
	EE MetaMax [SD] (kcal/h)	245 (±64)	278 (±68)	212 (±42)
	RMSE (kcal/h)	108	130	81
	MAPE (%)	38	39	37
	Pearson *r*	0.29	$-0.47^{\dagger }$	0.96**
Up Fast	n	9	4	5
	Mean Diff [95% CI] (kcal/h)	*-10* [*-154*, 133]	24 [*-389*, 437]	*-38* [*-199*, 123]
	*p*-value	0.871	0.864	0.547
	LLA [95% CI] (kcal/h)	*-375* [*-630*, *-121*]	*-484* [*-1263*, 295]	*-292* [*-588*, 5]
	ULA [95% CI] (kcal/h)	355 [100, 609]	533 [*-246*, 1311]	216 [*-81*, 512]
	Mean Predicted [SD] (kcal/h)	594 (±115)	668 (±78)	535 (±110)
	EE MetaMax [SD] (kcal/h)	584 (±199)	692 (±218)	497 (±150)
	RMSE (kcal/h)	176	226	122
	MAPE (%)	22	24	20
	Pearson *r*	0.40	$-0.41^{\dagger }$	0.54

** *p < 0.01*; $^{\dagger }$ interpret with caution (*n łe 6*). $^{\ddagger }$ One participant (P2) excluded from the Down phase due to implausible MetaMax reference values (mean actual EE: 85.9 kcal/h, range: 4.1–578.3 kcal/h). Results including P2 for completeness: MAPE *=* 65%, RMSE *=* 189 kcal/h, mean difference *=* *-132* kcal/h (*p~=~0.064*). A negative mean difference indicates sensor overestimation (Reference − Predicted *< 0*). Subgroup analyses are exploratory due to small sizes (*n = 4*–7). Marginal and Conditional $R^2$ were omitted from validation results; participant-level Pearson *r* is reported instead.

## Discussion

The present study investigated the potential of in-ear HR and ACC measurements from the $^\circ$One and $^\circ$Two sensors for predicting activity EE. Linear mixed-effects models were developed using a strict 30-second steady-state window (150–180 s) from each laboratory exercise stage, ensuring calibration was based on stabilised oxygen consumption and heart rate data.

By incorporating participant-specific random intercepts, the models accounted for inter-individual metabolic variability, which explained a significant portion of total variance (conditional $R^2_c$ up to 0.95) in the calibration phase. When applied to outdoor walking conditions, the models showed no significant systematic bias (*p> 0.05*) across all test phases, suggesting acceptable group-level agreement. However, prediction accuracy was variable across conditions and subgroups, and results should be interpreted within the context of the study’s limitations, including small sample size and the absence of downhill data in model training.

### Predicting energy expenditure

For both sensors, the model demonstrating the highest explanatory power during calibration integrated HR, ACC, and biological sex, achieving marginal $R^2_m$ values of 0.79 and 0.80 for $^\circ$One and $^\circ$Two respectively. When accounting for inter-individual metabolic variability through random intercepts, the conditional $R^2_c$ reached 0.92 for $^\circ$One and 0.95 for $^\circ$Two in the calibration phase, indicating that the mixed-effects framework captured a large proportion of variance in metabolic demand during the laboratory test. This result aligns with previous studies emphasising the superiority of combining HR and accelerometry over single-sensor inputs^18,19,37^.

The models were trained on laboratory uphill treadmill data and applied to an outdoor setting that included downhill walking, a condition in which the participant’s head faces downward, affecting the ear-worn accelerometer orientation. For the Down walking condition, the sensors showed a non-significant mean difference (*p> 0.05*) compared to the MetaMax 3B, suggesting the random intercept structure may have partially compensated for the postural and biomechanical differences of eccentric downhill locomotion. However, this interpretation is speculative, and dedicated training data for downhill conditions would be needed to verify this mechanism.

Overall, the cosinuss$^\circ$ in-ear sensors $^\circ$One and $^\circ$Two demonstrated MAPE of 22% and 25%, respectively, during comfortable-pace uphill walking. A recent study reported MAPE for several commercial devices under similar conditions, including the Fitbit Sense (45.1%), Polar Vantage V (15.6%), and Apple Watch 6 (24.1%)^45^. These findings indicate that the performance of the cosinuss$^\circ$ in-ear sensors is broadly comparable to some commercial activity trackers for comfortable-paced walking.

For comparison with the LeBoeuf et al^46^. study, which reported an $R^2$ of 0.86 using a simple linear model without random effects, the appropriate comparison from our study is the marginal $R^2_m$ rather than the conditional $R^2_c$. Our marginal $R^2_m$ values during outdoor validation ranged from 0.26 to 0.66 for $^\circ$One and from 0.28 to 0.43 for $^\circ$Two, which are lower than the LeBoeuf values, likely reflecting the added complexity of uncontrolled outdoor terrain compared to a fixed treadmill protocol.

### Differences Between male and female participants

The analysis of predicted EE demonstrated that female participants exhibited lower energy expenditure than male counterparts across uphill conditions, while demonstrating comparable EE levels during descent. This finding partially contrasts with Browning et al^47^., who suggested that normal-weight women may have relatively higher net metabolic rates during walking compared to normal-weight men, attributed to lower standing metabolic rates and reduced lean body mass^48,49^.

However, walking speed was not controlled or measured during the outdoor tests. The observed sex differences in EE may therefore reflect differences in self-selected walking speed rather than physiological factors alone, particularly during the Up Fast phase where participants were instructed to walk as fast as possible. It is therefore plausible that the sex coefficient in the calibration model partially reflects differences in body mass and self-selected walking speed between male and female participants, rather than a direct biological sex effect; future models should incorporate body mass and walking speed as explicit covariates. Future studies should monitor walking speed to allow proper attribution of sex-based EE differences.

Pearson correlation coefficients indicated stronger correlations for female participants in the Up and Down conditions. Male subgroup correlations were weak or negative across most conditions for both sensors, which limits conclusions about individual-level prediction accuracy for male participants. This finding likely reflects the very small male subgroup sizes (n *= 3*–6) in the outdoor validation, which resulted in statistically unstable correlation estimates. The overall non-significant Bland-Altman biases for both sexes indicate acceptable group-level performance, but individual-level accuracy requires improvement and larger validation studies before clinical use.

It is also important to note that the non-significant mean differences in Bland-Altman analysis do not imply equivalence between predicted and reference EE. They indicate only that the systematic bias was not statistically distinguishable from zero, which is partly a function of the small sample sizes and wide limits of agreement.

### Comparison between sensors °One and °Two

In this study, two different cosinuss$^\circ$ in-ear sensors were used. The main difference is the LED wavelength: green (520 nm) for $^\circ$One and red/infrared (665/940 nm) for $^\circ$Two. Sensor $^\circ$Two showed a slightly higher marginal explanatory power during calibration ($R^2_m = 0.80$) compared to $^\circ$One ($R^2_m = 0.79$).

To assess HR measurement accuracy directly, HR from both sensors was compared against the Polar H7 chest strap reference using steady-state laboratory data. Sensor $^\circ$One showed a mean difference of *-0.13* bpm, RMSE of 9.25 bpm, and Pearson *r = 0.964* (*p < 0.001*) against the Polar H7. Sensor $^\circ$Two showed a mean difference of *-0.73* bpm, RMSE of 9.48 bpm, and Pearson *r = 0.960* (*p < 0.001*). Both sensors demonstrated comparable and high agreement with the reference HR. The marginally higher EE model performance of $^\circ$Two during calibration may reflect the deeper tissue penetration characteristics of red/infrared light at this anatomical site, but the HR accuracy data do not indicate a meaningful difference between sensors in this regard.

It should be noted that in the $^\circ$Two calibration model, only HR reached statistical significance (*p < 0.001*), while ACC (*p = 0.233*) and Sex (*p = 0.440*) did not. This contrasts with the $^\circ$One model, where both HR (*p < 0.001*) and ACC (*p = 0.001*) were significant. Future studies with larger samples should investigate whether ACC and Sex provide meaningful independent contributions to EE prediction for the $^\circ$Two sensor, or whether an HR-only model would be sufficient.

### Comparison with other devices

EE estimation MAPE from commercial wrist-worn wearables varied between 14% and 210% when walking, brisk walking, and jogging^50^. In another study, seven commercially available wrist-worn monitors showed MAPE values ranging from 27% to 93%^51^. The Polar Vantage has been reported to achieve a MAPE of 20.6%^52^.

The outdoor EE estimation MAPE values for the Up and Up Fast conditions in our study ranged from 22% to 26% for $^\circ$One and 22% to 25% for $^\circ$Two, broadly comparable to these commercially available devices. The Down condition showed notably higher MAPE (37%–38%), likely driven by the lower absolute EE during downhill walking amplifying percentage errors, and by the absence of downhill training data in the calibration models. Direct comparison of these MAPE values with studies using controlled treadmill speeds should be interpreted with caution, as self-selected outdoor walking speed introduces additional variability not present in laboratory-based validation protocols.

Concerning ear-worn sensors, Bouarfa et al^53^. reported that their predicted EE exhibited a 9% deviation from total EE as quantified by doubly labelled water. In our study, the deviation from reference EE across all participants and phases ranged from approximately 2% to 82% for $^\circ$One and from 2% to 246% for $^\circ$Two, with the highest individual deviations occurring in the Down phase. These wide ranges reflect the inter-individual variability and the limitations of applying a group-calibrated model to individual participants.

### Strengths and limitations

To the best of our knowledge, this is among the first studies demonstrating the potential of commercial, wearable, multimodal in-ear sensors for predicting EE using simultaneous HR and accelerometry. The protocol design allowed data collection and model training in controlled laboratory conditions, with application in an outdoor, ecologically valid setting.

One limitation was the relatively small and homogeneous sample. While biological sex was incorporated into the final $^\circ$One model, other anthropometric factors such as age, weight, and height were not included as separate predictors. The small male subgroup sizes in the outdoor validation (n *= 3*–6) resulted in statistically unstable correlation estimates, and conclusions about male-specific prediction accuracy should be treated as preliminary.

The outdoor validation sample of 12 to 13 participants is at the lower threshold for Bland-Altman analysis, which generally requires at least 30 to 40 observations for stable limits of agreement estimates^43^. The wide limits of agreement reported here, reaching ± 268 kcal/h for $^\circ$One and ± 354 kcal/h for $^\circ$Two in the Up Fast phase, should therefore be interpreted with particular caution and are likely to narrow substantially with larger samples.

Another limitation was that the model was trained exclusively on uphill treadmill data. The absence of downhill training data likely reduced prediction accuracy during outdoor downhill walking. Walking speed was not controlled or measured during the outdoor tests, which prevents attribution of sex differences in EE to physiological versus behavioural factors. Furthermore, direct comparison of MAPE values with studies using controlled treadmill speeds should be interpreted with caution, as self-selected outdoor walking speed introduces additional variability not present in laboratory-based validation protocols.

Additionally, the non-significant Bland-Altman bias should not be interpreted as equivalence between predicted and reference EE. The wide limits of agreement, particularly in the Up Fast phase (± 268 kcal/h for $^\circ$One and ± 354 kcal/h for $^\circ$Two), indicate that individual-level prediction accuracy remains insufficient for clinical application at this stage.

Future studies should implement downhill walking in model training, include repeated measurements with diverse free-living activities, control for walking speed, and recruit larger samples with greater diversity in age, body composition, and fitness level.

## Conclusion

In conclusion, this preliminary study investigated the feasibility of predicting EE from HR and ACC data recorded by commercial in-ear sensors, compared against indirect calorimetry using the MetaMax 3B. Under the described experimental conditions, both the $^\circ$One and $^\circ$Two sensors demonstrated preliminary group-level agreement during outdoor walking, with no significant systematic bias detected across phases. MAPE values for uphill walking conditions were broadly comparable to several commonly used commercial wrist-worn activity trackers.

However, several limitations must be acknowledged. Prediction accuracy was variable across conditions and subgroups, with notably higher MAPE during downhill walking and weak or negative Pearson correlations for male participants in several phases. The relatively small and homogeneous sample, the absence of downhill walking in model training, and the lack of walking speed measurement restrict the generalisability of these findings. Furthermore, outdoor predictions were generated using participant-specific random intercepts from the calibration LMM, meaning the outdoor testing phase reflects participant-calibrated performance rather than truly independent external validation. Fixed-effect-only prediction in an independent sample is required to establish genuine generalisability to new users, and represents the primary priority for follow-up work. Furthermore, non-significant Bland-Altman bias does not imply equivalence, particularly given the wide limits of agreement observed across conditions.

Based on these results, the in-ear sensors should be used with caution in clinical or sports settings for individual-level EE estimation. With further improvements in model training, larger validation samples, inclusion of diverse movement conditions, and walking speed monitoring, in-ear multimodal sensors could represent a promising tool for continuous EE monitoring in rehabilitation and sports medicine contexts, given their capacity for simultaneous measurement of multiple vital parameters.

With further validation in larger and more diverse cohorts, in‑ear sensing could support continuous energy expenditure monitoring in free‑living conditions, with potential applications in rehabilitation, sports training, and chronic disease management. The present findings should, however, be interpreted as a feasibility demonstration rather than immediate clinical guidance.

## Data Availability

The data presented in this study are available on request from the corresponding author.

## Abbreviations

EE: Energy expenditure
HR: Heart rate
ACC: Acceleration
MET: Metabolic
BMI: Body mass index
MAPE: Mean absolute percentage error
RMSE: Root mean square error
CI: Confidence interval
$\dot{V}$CO$_2$: Carbon dioxide production
$$\dot{V}$$O$$_2$$: Oxygen consumption
$\dot{V}$O$_{2\text {max}}$: Maximal oxygen uptake
LMM: Linear mixed-effects
PPG: Photoplethysmography
ECG: Electrocardiography
LoA: Limits of agreement
